# A rare combined injury of the cruciate ligament and occult tears of the medial and lateral heads of the gastrocnemius muscle: A case report

**DOI:** 10.1097/MD.0000000000043742

**Published:** 2025-08-08

**Authors:** Dazhi Li, Hui Ma, Lei Wang, Xiaodong Wu, Yanming Li

**Affiliations:** a School of Clinical Medicine, Jining Medical University, Jining, Shandong Province, China; b Department of Joint Surgery, Affiliated Hospital of Jining Medical University, Jining, Shandong Province, China; c Department of Spine Surgery, Affiliated Hospital of Jining Medical University, Jining, Shandong Province, China.

**Keywords:** anterior and posterior cruciate ligaments torn, closed injury, knee hyperextension, lateral and medial heads of the gastrocnemius muscle torn, physical examination under anesthesia

## Abstract

**Rationale:**

The diagnosis and treatment of high-energy knee trauma primarily focus on ligamentous and bony injuries, often overlooking potential concomitant gastrocnemius muscle injuries. Isolated closed tears of the medial and lateral heads of the gastrocnemius muscle (without associated fractures or dislocations) are exceptionally rare and clinically insidious, increasing the risk of missed diagnosis and compromised patient outcomes. This article presents a novel case of combined cruciate ligament injury and occult tears of both gastrocnemius heads, a condition not previously documented in the literature.

**Patient concerns:**

A 53-year-old male was injured by a car and suffered a wound on his left knee. He presented with obvious symptoms of pain, swelling, and limited mobility.

**Diagnoses:**

The physical examination showed positive Lachman test, anterior and posterior drawer tests, and pivot shift test. Resistance to plantar flexion was weak. The floating patella sign was positive. The McMurray test was suspiciously positive. Combined with magnetic resonance imaging (MRI), the diagnosis was anterior and posterior cruciate ligament injuries of the left knee joint. After the anesthesia, the patient was reexamined. The result showed that the left knee joint was obviously overextended. Eventually, it was confirmed as anterior and posterior cruciate ligament injuries combined with rupture of the medial and lateral heads of the gastrocnemius muscle.

**Interventions:**

A staged surgical approach was employed: primary posterior capsular and gastrocnemius repair, followed by secondary arthroscopic anterior and posterior cruciate ligament reconstruction.

**Outcomes:**

Six months after discharge from the hospital, the patient underwent a reexamination with MRI, the results were satisfactory. The patient was able to walk without pain and regain mobility. There was no sensation of instability in the knee joint.

**Lessons:**

This case highlights that isolated gastrocnemius tears are diagnostically challenging but must be considered in hyperextending knees, examination under anesthesia enhances detection of occult instability, while MRI is a valuable diagnostic tool, its interpretation should be integrated with clinical findings to ensure that subtle soft tissue injuries are not overlooked.

## 1. Introduction

Knee ligament injuries frequently occur in conjunction with damage to surrounding musculoskeletal and neurovascular structures.^[1]^ High-energy trauma mechanisms, particularly those involving knee hyperextension with concurrent gastrocnemius contraction, can generate excessive tensile forces on the medial head of the gastrocnemius. In severe cases, this may result in tibial plateau avulsion fractures.^[2]^ As demonstrated by Park et al, posterior cruciate ligament injuries commonly involve concomitant posterior capsular damage,^[3]^ reflecting the intimate anatomical relationship between the joint capsule and gastrocnemius tendon origins. While magnetic resonance imaging (MRI) serves as the primary diagnostic modality for evaluating these injuries, its limited sensitivity for detecting subtle muscular pathology may lead to interpretive challenges.^[4]^

It is worth noting that closed tears of the medial and lateral heads of the gastrocnemius muscle (without accompanying fractures or dislocations) are extremely rare in clinical practice. Such injuries pose diagnostic challenges due to their overlapping clinical manifestations with numerous common knee joint pathologies. This article reports the first case of closed tears of the medial and lateral heads of the gastrocnemius muscle without fractures or dislocations. A deeper understanding of the clinical features, diagnostic methods, and treatment strategies of such rare injuries is of great significance for improving the accuracy of diagnosis and treatment.

## 2. Case report

### 2.1. Current medical history and physical examination

Patient Lv Mou, male, 53 years old, visited the Affiliated Hospital of Jining Medical University on November 13, 2024 due to “left knee pain and swelling after trauma with limited mobility for 2 days.” Two days before admission, the patient was hit by a car while riding a bicycle. After the injury, the patient felt persistent dull pain in the left knee, intermittent sharp pain, the pain was aggravated upon pressing, the knee was significantly swollen, and mobility was restricted. There were extensive subcutaneous bruises, no open wounds, no bleeding, no coma, no loss of consciousness or limb numbness, no palpitations, chest tightness, breathing difficulties, and no incontinence of urine or stool. Physical examination: the left knee joint was significantly swollen, with normal skin temperature. There was tenderness around the knee joint, more pronounced on the medial side. The pivot shift test was positive, the Lachman test was positive, the anterior and posterior drawer tests were positive, the McMurray test was suspiciously positive, the floating patella sign was positive, and resistance plantar flexion was weak. The Thompson test was negative, and the internal and external rotation stress tests were negative. The range of motion of the knee joint was 100° flexion and 0° extension. The blood supply and sensation of the lower extremity were normal.

### 2.2. Ancillary examinations

*MRI findings*: soft tissues around the left knee joint are swollen, with patchy low signal on T1WI and high signal on PDWI-FS: the edges of the bones of the various joint components in the left knee joint are sharp and show a lip-like change, the articular cartilage is locally thinned, and patchy low signal on T1WI and slightly higher signal on PDWI-FS can be seen under the joint surface bone. The anterior and posterior cruciate ligaments are swollen, and the fibrous bundles are not continuous: the morphology of the bilateral collateral ligaments is slightly swollen, and a small amount of low signal on T1WI and high signal on PDWI-FS can be seen inside. The posterior horn of the medial meniscus and the anterior horn of the lateral meniscus show strip-like low signal on T1WI and high signal on PDWI-FS, and the local area communicates with the joint cavity: the posterior horn of the lateral meniscus shows strip-like low signal on T1WI and high signal on PDWI-FS. The left knee joint cavity and the suprapatellar bursa can show arc-shaped low signal on TIWI and high signal on PDWIFS fluidic signals.

**Figure 1.**
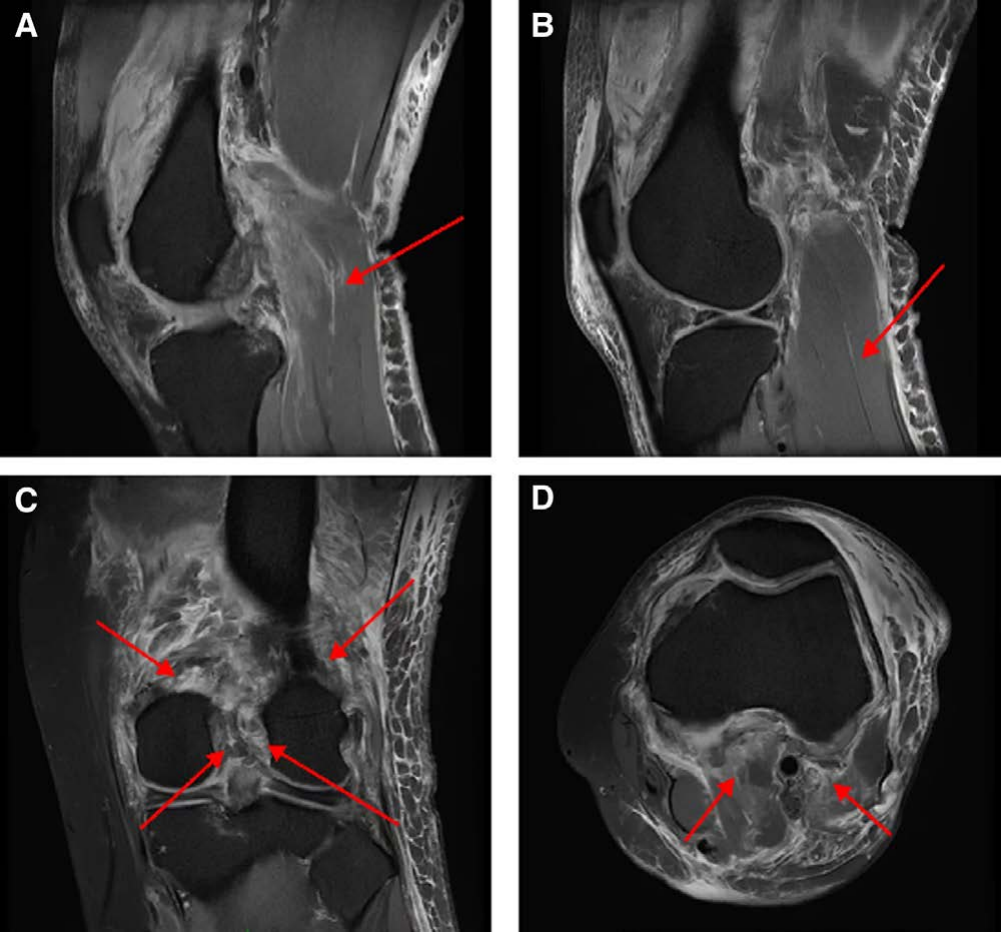
Proton density magnetic resonance images 2 days after the car accident, (A) sagittal image of the ruptured medial head of the gastrocnemius muscle (arrow), (B) sagittal image of the torn lateral head of the gastrocnemius muscle (arrow), (C) coronal images of the ruptured medial head, lateral head, posterior cruciate ligament and anterior cruciate ligament of the gastrocnemius muscle (arrow, from top to bottom, from left to right), (D) axial images of the ruptured medial head and lateral head of the gastrocnemius muscle (arrow, from left to right).

*MRI diagnosis*: 1. soft tissue injury of the left knee joint, and it cannot be ruled out that the anterior and posterior cruciate ligaments of the left knee joint are ruptured; 2. the posterior horn of the medial meniscus and the anterior horn of the lateral meniscus of the left knee joint are torn; 3. osteoarthritis and bone cartilage injury of the left knee joint; 4. local bone contusion of the left knee joint needs to be excluded; 5. chronic injury of the bilateral collateral ligaments of the left knee joint; 6. injury of the posterior horn of the lateral meniscus of the left knee joint (degree II); 7. synovial fluid in the left knee joint (Fig. 1).

### 2.3. Admission diagnosis

Injury to the anterior cruciate ligament and posterior cruciate ligament of the left knee, injury to the meniscus of the left knee, chronic injury to the bilateral collateral ligaments of the left knee; osteoarthritis of the left knee, cartilage injury of the left knee joint, effusion in the left knee.

### 2.4. Treatment process

**Figure 2.**
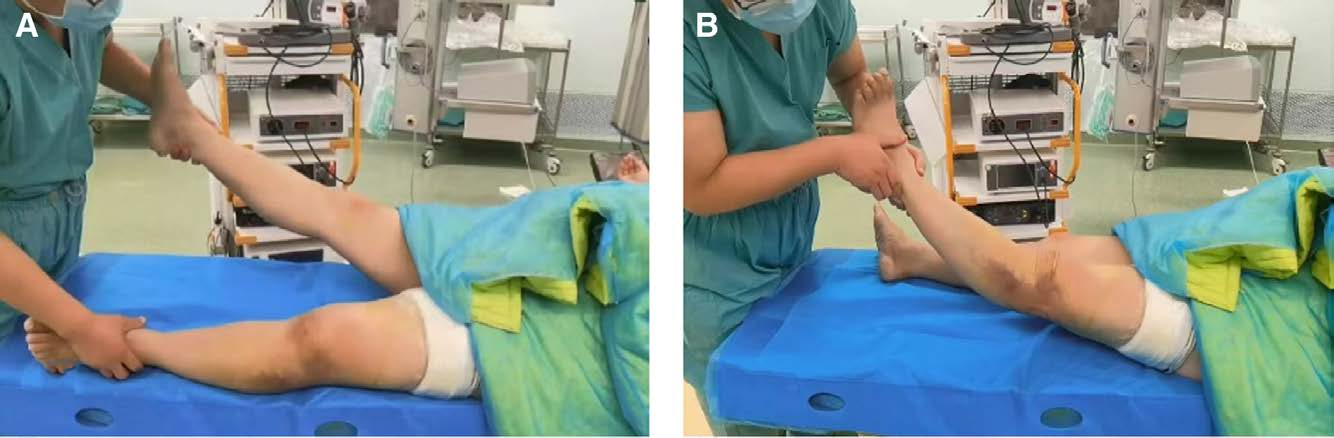
Physical examination after anesthesia: (A) normal side, (B) affected side, knee is significantly hyperextended > 15°.

The initial surgical plan for anterior and posterior cruciate ligament reconstruction was reconsidered following an examination under anesthesia that demonstrated >15° of left knee hyperextension (Fig. 2), a finding inconsistent with isolated cruciate ligament injuries and indicative of significant posterior structural compromise. MRI reevaluation confirmed complete avulsion of both medial and lateral gastrocnemius heads from their femoral condylar attachments, revealing a complex injury pattern involving the gastrocnemius muscle complex and posterior joint capsule. Given the documented poor outcomes of conservative management for such injuries and the high failure risk associated with performing cruciate ligament reconstruction without first addressing posterior instability, we determined that restoration of the primary static stabilizers through staged surgical intervention was imperative to achieve joint stability.

**Figure 3.**
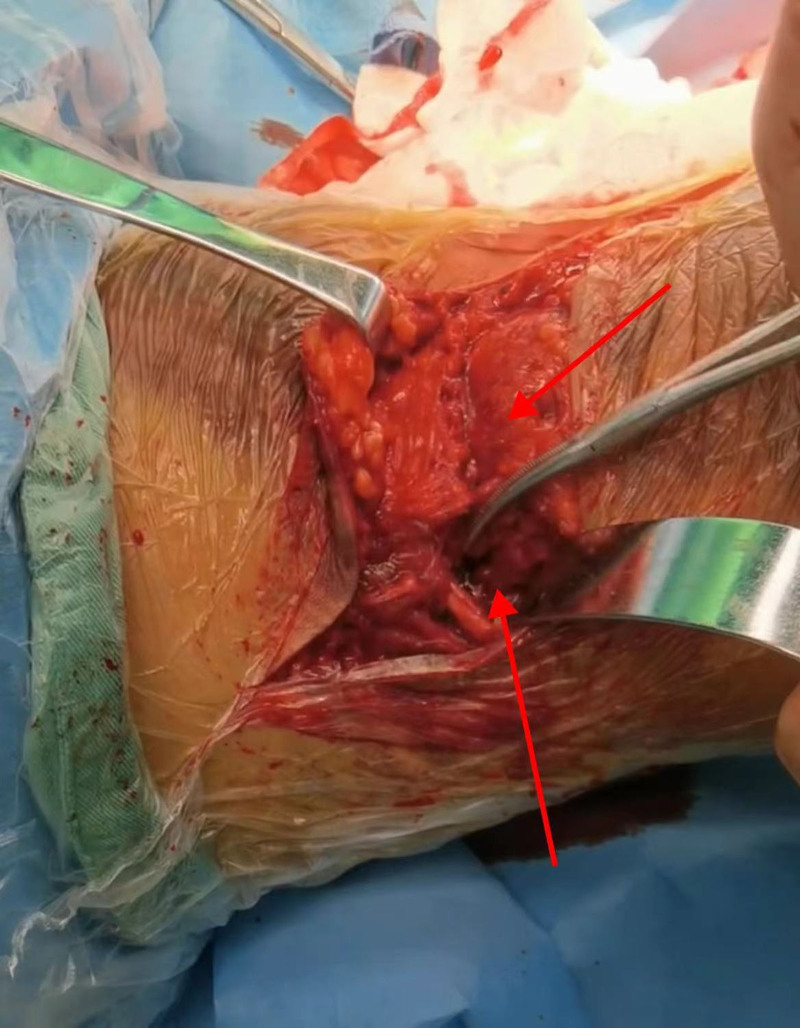
During the operation, the internal and external heads of the gastrocnemius muscle that were ruptured could be observed (arrows, from top to bottom).

The patient was placed in a prone position. The skin of the left lower limb surgical area was routinely disinfected, and sterile surgical towels were laid out and a protective skin membrane was attached. A large S-shaped incision was made on the bruised area of the skin at the popliteal fossa of the left knee, with the incision approximately 15 cm long, starting 5 cm proximally from the popliteal crease, and extending outward and downward along the skin folds of the popliteal fossa to the medial side of the fibular head. After layer-by-layer incision of the skin and subcutaneous tissue, extensive subcutaneous congestion and hematoma formation were observed, with a volume of approximately 50 mL. During the operation, it was found that the popliteal fascia was completely transected and the ends retracted about 3 cm; the small saphenous vein and its branches (medial and lateral dural veins) were completely ruptured; the lateral head of the gastrocnemius muscle was torn off from the posterior side of the medial condyle of the femur, and the lateral head was torn off from the lateral condyle (with partial muscle fiber tearing); the posterior joint capsule of the knee joint was longitudinally torn (about 4 cm long). After careful examination, it was confirmed that the main vessels (popliteal arteries and veins) and branches of the sciatic nerve (tibial nerve and common peroneal nerve) in the popliteal fossa and the structure of the sciatic nerve branches were intact, with no obvious damage (Fig. 3).

*Surgical handling process*: the wound was thoroughly rinsed and hemostatic, with attention paid to protecting the important vessels and nerve structures in the popliteal fossa. The small saphenous vein and its branches that were broken were ligated with silk thread, and the broken peroneal lateral skin nerve did not need to be treated. The posterior knee joint capsule was sutured intermittently, and additional strengthening suturing was performed on the lateral side. The peroneal muscle medial and lateral heads were sutured and fixed to the femoral condyle attachment point with silk thread, and the repair points of the medial and lateral heads were reinforced with suturing. The popliteal fascia, subcutaneous tissue, and skin were repaired intermittently, and the incision was covered with sterile dressings for bandaging. The left lower limb was fixed in a 20° flexion position with a plaster splint. After a 3-month recovery period, the patient’s knee joint stability improved significantly, and the excessive extension symptoms were relieved. The patient ultimately successfully underwent arthroscopic reconstruction surgery for the anterior and posterior cruciate ligaments.

### 2.5. Follow-up and functional assessment

At the 3-month postoperative follow-up, significant functional improvement was demonstrated by an increase in the AOFAS score from 45 (preoperative) to 70 points, with concomitant reduction of hyperextension to 5°.

**Figure 4.**
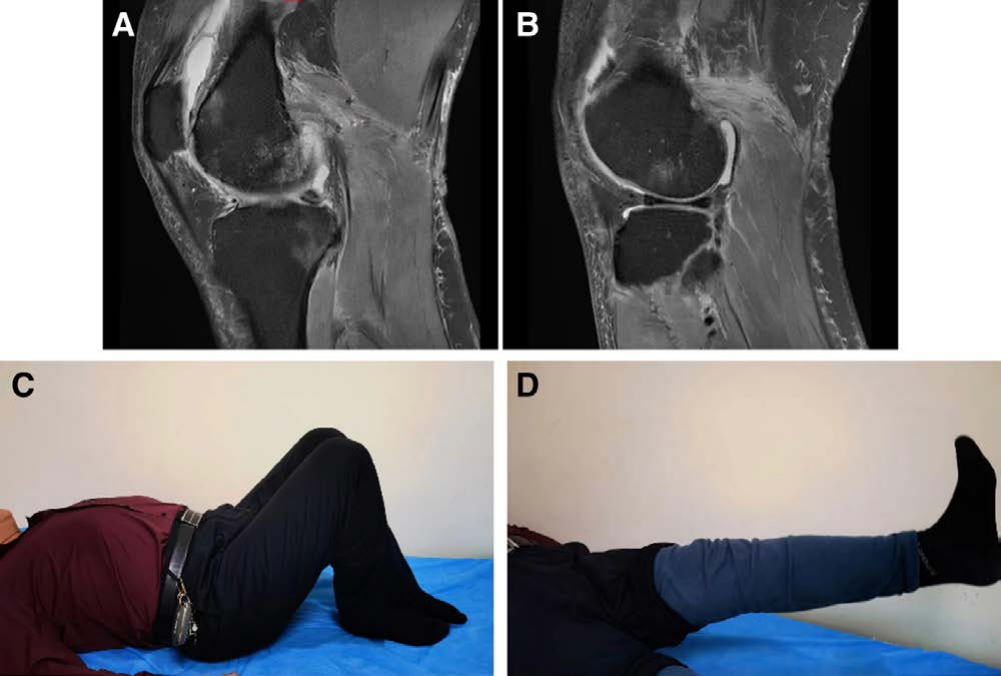
Follow-up examination 6 months after the first-stage surgery: (A) sagittal proton density magnetic resonance image of the medial head of gastrocnemius muscle (arrow), (B) sagittal proton density magnetic resonance image of the lateral head of gastrocnemius muscle (arrow), (C and D) the patient’s left knee hyperextension was significantly improved.

By 6 months postoperatively, the patient had achieved complete gait normalization. MRI evaluation confirmed excellent healing of posterior knee structures (Fig. 4), supported by outstanding functional outcomes: Lysholm score of 90 points, Tegner activity level of 6, and KT-1000 arthrometer measurements showing only 2mm side-to-side difference in anterior–posterior translation. Clinical examination revealed complete resolution of instability, with negative Lachman, anterior/posterior drawer, and pivot shift tests, indicating successful restoration of knee stability.

## 3. Discussion

The gastrocnemius muscle consists of 2 distinct origins: the medial head arises from the posterior aspect of the medial femoral condyle, while the lateral head originates from the corresponding region of the lateral femoral condyle. These 2 muscular heads converge to form a unified muscle belly, which subsequently combines with the deep-lying soleus tendon in the mid-calf region to constitute the exceptionally robust Achilles tendon, ultimately inserting at the calcaneal tuberosity.^[5]^ Clinically, the majority of gastrocnemius injuries occur at the myotendinous junction, with the medial head demonstrating greater vulnerability to trauma due to its larger cross-sectional area and increased biomechanical load during functional activities.^[6]^ In cases of closed knee joint injuries, it is extremely rare to observe simultaneous rupture of the medial and lateral heads of the gastrocnemius muscle without concurrent fractures or joint dislocation.

Early clinical evaluation of traumatic knee injuries frequently fails to identify the full spectrum of soft tissue damage.^[7]^ Our case initially demonstrated classic signs of bicruciate ligament injury, including post-traumatic knee pain, restricted mobility, and positive instability tests (Lachman, anterior/posterior drawer, and pivot shift tests). As with most knee instability procedures, examination under anesthesia proved crucial for both confirming the preoperative diagnosis and revealing additional pathology.^[8]^ However, there is a risk of false negative in physical examination when the patient is awake. Our patient showed severe and hidden hyperextension of the knee joint under muscle relaxation. This critical finding highlights the essential diagnostic principle that clinicians must maintain a high index of suspicion for uncommon injury patterns even when evaluating patients with classic presentations of frequent knee pathologies. The clinical manifestations of gastrocnemius and posterior capsular injuries demonstrate significant overlap with several more prevalent conditions, including cruciate ligament ruptures, tibial plateau avulsion fractures, Achilles tendon tears, and deep venous thrombosis. A comprehensive diagnostic approach, incorporating both meticulous history-taking and methodical physical examination techniques, serves as the fundamental prerequisite for accurate differential diagnosis in such complex cases.^[9]^ While MRI serves as the cornerstone imaging modality for knee trauma evaluation, its diagnostic accuracy remains limited in assessing the intricate posterior knee anatomy. The close anatomical relationship between the gastrocnemius muscle heads and posterior joint capsule, combined with acute inflammatory changes (edema and hemorrhage), frequently creates imaging artifacts that obscure subtle soft tissue injuries. In the present case, these technical limitations initially diverted diagnostic attention toward the more obvious anterior and posterior cruciate ligament tears, while masking the concomitant gastrocnemius avulsions. Such diagnostic oversights, stemming from imaging interpretation challenges, may significantly compromise treatment efficacy and long-term functional outcomes if these critical posterior stabilizers remain unaddressed.^[7]^ Therefore, clinicians should carefully interpret the images and actively conduct imaging studies. De Smet et al emphasized that precise imaging diagnosis must be combined with detailed clinical assessment. For suspected cases, thin-slice sagittal MRI scans are recommended. Surgery exploration should be carried out immediately if necessary.^[9]^

The posterior joint capsule of the knee joint is an important structure for maintaining the stability of the knee joint. The tendons and ligaments attached to the joint capsule provide reinforcement for it.^[3,4,8]^ The research conducted by Noyes et al indicates that the posterior medial and posterior lateral joint capsule structures are the main resistances preventing knee hyperextension.^[10]^ When the knee joint is subjected to excessive force, obvious knee hyperextension may occur, which suggests that there might be damage to the gastrocnemius muscle and the posterior joint capsule.^[4,11]^ Therefore, a thorough examination and assessment of the knee joint is of vital importance. Research indicates that hyperextension of the knee joint is a risk factor for the failure of ligament reconstruction,^[12]^ if direct cross-ligament reconstruction is performed, weakness of the gastrocnemius muscle may lead to excessive stress on the graft, increasing the risk of surgical failure. A staged surgical approach, where joint capsule repair and gastrocnemius muscle repair are carried out first, followed by cross-ligament reconstruction in the second stage, can provide a more favorable biomechanical foundation for the graft. Therefore, for patients with ligament injuries combined with knee hyperextension, staged surgery may be a safer and more effective treatment strategy.^[8,13]^

## 4. Conclusion

In conclusion, for patients with closed knee joint injuries, the combination of ligament injury and rupture of the medial and lateral heads of the gastrocnemius muscle (without fracture or dislocation) is extremely rare in clinical practice. It is necessary to conduct routine assessment of the stability of the knee joint after anesthesia. A significant and obvious knee hyperextension with a large angle should consider the possibility of combined rupture of the gastrocnemius muscle and the posterior joint capsule of the knee. Comprehensive assessment of MRI imaging features is an important diagnostic basis. In necessary cases, intraoperative exploration is required.

## Acknowledgments

The authors sincerely thank colleagues in this department as well as the patients for their selfless dedication. At the same time, they are grateful to the patients or their close relatives for signing the informed consent form.

## Author contributions

**Conceptualization:** Lei Wang, Xiaodong Wu.

**Writing – original draft:** Dazhi Li, Hui Ma.

**Writing – review & editing:** Yanming Li.
